# Targeted drug combination therapy design based on driver genes

**DOI:** 10.18632/oncotarget.26985

**Published:** 2019-09-03

**Authors:** Lilian Zsákai, Anna Sipos, Judit Dobos, Dániel Erős, Csaba Szántai-Kis, Péter Bánhegyi, János Pató, László Őrfi, Zsolt Matula, Gábor Mikala, György Kéri, István Peták, István Vályi-Nagy

**Affiliations:** ^1^ Vichem Chemie Research Ltd., Budapest, Hungary; ^2^ Department of Pharmaceutical Chemistry, Semmelweis University, Budapest, Hungary; ^3^ Department of Hematology and Stem Cell Transplantation, Central Hospital of Southern Pest National Institute of Hematology and Infectious Diseases, Budapest, Hungary; ^4^ Oncompass Medicine Hungary Ltd., Budapest, Hungary; ^5^ Department of Pharmacology, Semmelweis University, Budapest, Hungary; ^6^ MTA-SE Patho-Biochemistry Research Group, Department of Medical Chemistry, Semmelweis University, Budapest, Hungary; ^#^ Author deceased

**Keywords:** combination therapy, cancer driver gene, primary culture, small molecule compound, multiple myeloma

## Abstract

Targeted therapies against cancer types with more than one driver gene hold bright but elusive promise, since approved drugs are not available for all driver mutations and monotherapies often result in resistance. Targeting multiple driver genes in different pathways at the same time may provide an impact extensive enough to fight resistance. Our goal was to find synergistic drug combinations based on the availability of targeted drugs and their biological activity profiles and created an associated compound library based on driver gene-related protein targets. In this study, we would like to show that driver gene pattern based customized combination therapies are more effective than monotherapies on six cell lines and patient-derived primary cell cultures.

We tested 55–102 drug combinations targeting driver genes and driver pathways for each cell line and found 25–85% of these combinations highly synergistic. Blocking 2–5 cancer pathways using only 2–3 targeted drugs was sufficient to reach high rates of tumor cell eradication at remarkably low concentrations.

Our results demonstrate that the efficiency of cancer treatment may be significantly improved by combining drugs against multiple tumor specific drivers.

## INTRODUCTION

The definition and nature of driver genes have been discussed intensely in the last decade. Here, for the sake of simplicity, we highlight a manageable number of cancer driver genes as therapeutic targets based on the 138 driver genes identified by B. Vogelstein and his group and treat these genes as common feature points of multiple cancer genome mutational landscapes, a kind of focus that is critical for targeted drug development [1–5].

Since the majority of targeted therapies address only one oncogene, resistance develops eventually even if one of the actual drivers was targeted. The rapid development of drug resistance is due to the fact that more than a single driver may exist in a given tumor [6–8]. In addition, all tumor types are heterogeneous and certain cancer cell subpopulations or subclones tend to persist [9–11]. After the initial shock caused by the inhibition of a single key driver other non-targeted drivers come to the forefront. They are activated via feedback loops and/or with increased expression at the protein level and sometimes newly acquired mutations within the already mutated genes or in others [12–15]. All tumors with multiple driver mutations in their genome have the potency to show primary resistance to monotherapies therefore we need to target multiple drivers with combination therapies to overcome resistance [16–23]. The driver mutation profile of a tumor of an individual patient would be a valuable primary basis for a more precise and cost-effective approach of combination therapy design than combinatorial methods [26–29].

Here we present sequencing-based, targeted combination therapy studies on colon, lung and multiple myeloma cell lines and also on patient-derived surviving cultures in order to underpin the potency of the individual driver gene pattern based combinational method. We also would like to propose that the pursuit for simplicity may offer highly efficient approaches.

## RESULTS

### Targeting drivers in multiple myeloma cell line models

Multiple myeloma is a currently incurable hematological tumor with several well characterized cell line models [30–32]. In the case of RPMI8226, U266 and LP1 myeloma cell lines our targeted drug design was based on data from the Multiple Myeloma Cell Line Characterization Project [24] and the COSMIC database [25]. As a subsequent step, we eliminated the mutations that did not manifest in amino acid changes. Novel unknown mutations that lead to actual amino acid changes were considered structural changes that may result in functional alterations, therefore they are potential molecular targets that can be inhibited by specific pharmaceuticals. Frameshift mutations were automatically regarded as total function loss. By using this simple approach we identified the potential drivers and their associated targets for each myeloma cell line (Table 1).

**Table 1:** Driver genes and the associated driver targets of multiple myeloma cell lines

RPMI8226		U266		LP1	
Drivers	Target(s)	Drivers	Target(s)	Drivers	Target(s)
KDM6A	KDM6A, SETD2	KDM6A	KDM6A, SETD2	KDM6A	KDM6A, SETD2
ARID1A	AKT, PI3K, mTOR, AR	ARID1A	AKT, PI3K, mTOR, AR	CASP8	DNMT1, DNMT3A, HDAC
MAP3K1	MEK, AR	MAP3K1	MEK, AR	MAP3K1	MEK, AR
ARID1B	AKT, PI3K, mTOR, AR	ARID1B	AKT, PI3K, mTOR, AR	ARID1B	AKT, MTOR, AR
MLL3	HDAC, proteasome	MLL3	HDAC, proteasome	MLL3	HDAC, proteasome
NOTCH1	NOTCH	NOTCH1	NOTCH	CDKN2A	CDKs
KRAS	KRAS, Ftase, MEK	KRAS	KRAS, Ftase, MEK	TP53	HDAC, AURKA, MYC, CDKs, BCL2
TP53	CDKs, MYC, HDAC, AURKA, BCL2, PLK1	TP53	CDKs, MYC, HDAC, AURKA, BCL2, Topoisomerase II		
JAK3	JAKs, AR	JAK3	JAKs, AR		

Drivers were identified according to Vogelstein’s driver gene list based on the whole-genome sequencing data from the Multiple Myeloma Cell Line Characterization Project and the COSMIC database for mutational analysis. Targets that belong to the drivers were selected after manual literature mining.

The three cell lines share many common tumor suppressor genes (TSGs) with at least one loss-of-function mutation: KDM6A, ARID1A (in LP1, ARID1A’s partner, ARID2B is mutated), MAP3K1, TP53 and MLL. RPMI8226 and U266 have some other mutated drivers in common (e.g., KRAS, JAK3, NOTCH and CDH1).

Although IC_50_ values have been typically utilized for the characterization of the efficacy of a given compound, here we introduce the IC_95_ values and the corresponding Combination Indices (CI_95_s), because we found that these values are more informative when the research objective is to decrease the cancer cell number as much as possible. The range of combinational doses were chosen in a way that using the highest concentrations no detectable amount of living cells remained and then used the same curve fitting method as in the case of IC_50_ value determination, only had the cutoff at 5% viability instead of 50 when determining the IC_95_ value. CI_95_ values are derived from IC_95_ values in the same way as in the case of CI_95_ values. In this study, we considered a given drug combination effective based on two major factors: the IC_95_ value (which represents efficacy) and the CI_95_ value (which demonstrates the synergism or antagonism of the compounds when applied together).

IC and CI values for the myeloma cell lines can be found in Supplementary Table 1A–1C.

### Effect of driver-targeting compounds on the RPMI8226 cell line

We designed 75 combinations of 16 inhibitors. Mutated KRAS activity, which may be crucial in this system, was blocked by the farnesyl transferase inhibitor tipifarnib [33]. We used the pan-CDK inhibitor dinaciclib, being under investigation in clinical trials [34–36]. CUDC101 and CUDC907 are HDAC inhibitors. CUDC101 also inhibits EGFR, while CUDC907 has an effect on PI3Kα [23, 37, 38]. PF-03084014 is a gamma secretase inhibitor that blocks NOTCH signaling [39]. To block the androgen receptor (AR) we used an analogue of flutamide (Vichem Flutamide analogue) [40]. To target BCL2 we used GX15-070 (obatoclax) and ABT-263 [41–43]. We targeted the tankyrase system with the TRKA inhibitor AG879 and used Dp44mT as a topoisomerase II inhibitor [44, 45]. We used AGI-6780 to inhibit IDH1/2 and MG-132 to block the proteasome [46, 47]. MG-132 is one of the analogues of bortezomib, a promising therapeutic agent for multiple myeloma [48, 49]. To inhibit JAKs we used SB1317 which has also an effect against FLT3 [50]. Trametinib is an FDA-approved MEK inhibitor [51]. Finally, for FGFR inhibition, we used XL999, which inhibits FLT3, PDGFRs and VEGFR1-2-3 as well as FGFR1-2-3 [52] and an FGFR2–FGFR3-selective inhibitor developed by Vichem Ltd. (Vichem FGFR Inhibitor). For all 75 combinations, 50 were bicomponent, while 25 consisted of three different compounds. When CI_95_ was used as a basis to define synergism among two or three compounds we found that 48 out of 75 designed combinations (64%) were synergistic. Furthermore, 38 out of the 48 synergistic combinations (79%) had a CI value < 0.1 which means strong synergism. Regarding the ratio of the synergistic–non-synergistic combinations with respect to the number of drugs used, 26 out of 50 (52%) double combinations were synergistic and 22 out of 25 triple combinations were synergistic (88%). In Figure 2 we present a combination with high synergy as well as a low IC_95_ value.

**Figure 1:**
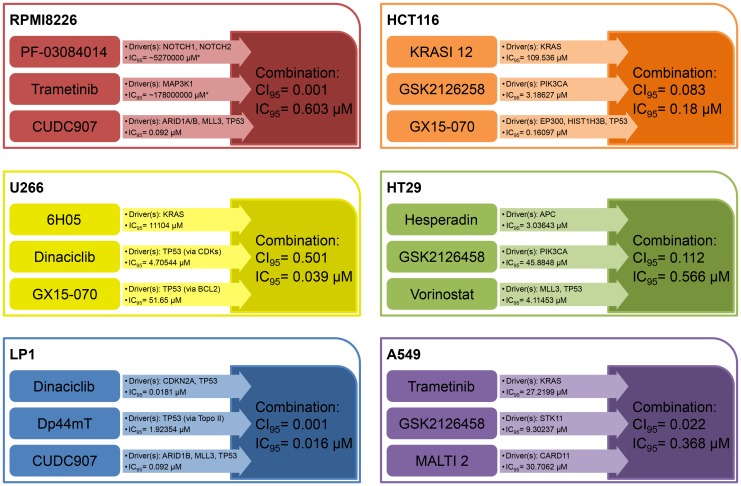
Selected synergistic combination examples for each cell line. Here we show one example of synergistic combination therapy for each cell line used. We applied IC_95_ values as an indicator of near-total effectiveness in killing tumor cells. CI_95_ values < 1 indicate synergism between drugs. Combinations are more effective than monotherapies with regard to the magnitude of the IC_95_ values and/or the number of drivers knocked out. Abbreviations: IC_95_: 95% cell killing; CI_95_: Combination Index associated with IC_95_; KRASI 12: KRAS Inhibitor 12; MALTI: MALT1 Inhibitor 2. ^*^Indicates that this value is a computational estimation which indicates that there’s no appreciable effect biologically.

**Figure 2:**
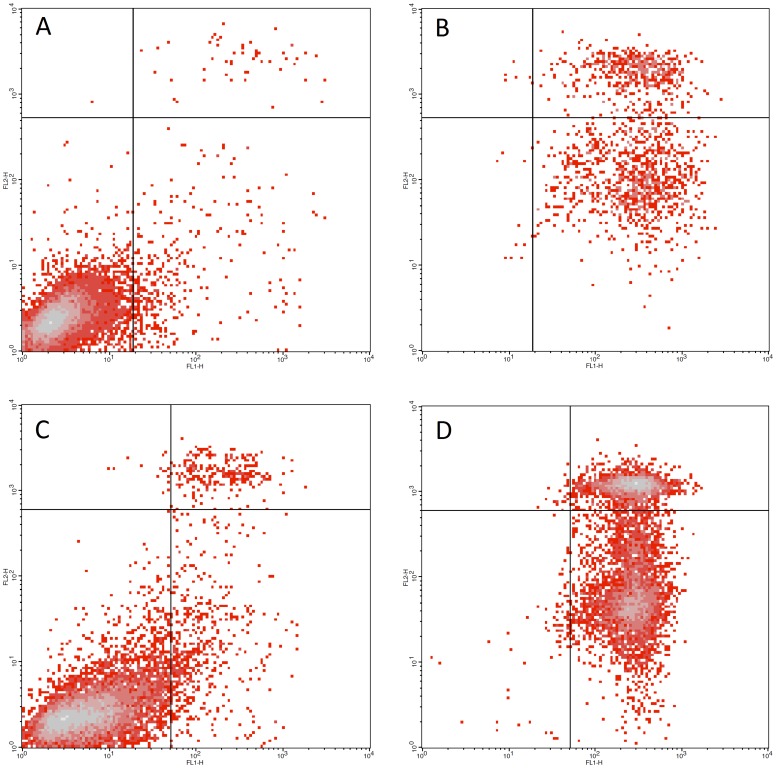
Low dose combination therapy effects on multiple myeloma cell lines. (**A**) LP1 cell line untreated control (**B**) LP1 cell line treated with 0.122 μM dinaciclib + MK2206 + CUDC907. 0.61% viable cells were detected (**C**) RPMI8226 cell line untreated control (**D**) RPMI8226 cell line treated with 0.122 μM dinaciclib + trametinib + PF-3084014. 1.26% viable cells were detected.

### Effect of driver-targeting compounds on the U266 cell line

Here we defined a highly similar set of driver genes and targets as on RPMI8226, however, U266 proved to be a much more resilient model system. To improve the effects on KRAS, proteasome and JAK targets, we incorporated compounds with different mechanisms of action or a slightly altered inhibitory profile and used the novel experimental KRAS inhibitor 6H05 [53], carfilzomib, a covalently binding bortezomib analogue [49], and XL019 (a more JAK-specific inhibitor) [54]. We also focused on the PI3K-AKT-mTOR pathway. We used three PI3K-mTOR inhibitors (GSK2126458 [55], XL765 [56] and PP242 [18]) and a highly selective allosteric AKT inhibitor, MK2206 [57–60]. We also used a pan-Aurora kinase inhibitor, Danusertib [61], and a PLK inhibitor, BI2536 [62, 63]. We targeted the Wnt pathway by inhibiting Porcupine with IWP-2 [64] and used a SET-SETD system-specific experimental inhibitor, NF279 [65]. We also used oltipraz, which interferes with the NF2 system by inhibiting NFE2L2 [66] with kinase inhibitors. Out of the designed 102 combinations, 74 combinations were double, and 28 were triple. According to CI_95_ value, 25 out of 102 combinations were synergistic (25%), 15 double (15%) and 10 triple (10%) combinations. Figure 1 depicts one of the successful combinations.

### Rates of synergistic combinations on the LP1 cell line

The LP1 cell line has some mutational qualities in common with the RPMI8226 and U266 lines. The ARID1B, CDKN1A and caspase 8 genes are mutant but the KRAS, NOTCH1, JAK and CDH1 genes are not. Out of the 86 designed combinations, 65 contained two and 21 had three drugs. We found that 48 combinations out of 86 were synergistic (56%) with close to 100% cell killing. 23 out of the 48 synergistic combinations (48%) had a CI value < 0.1. The ratio of synergistic to non-synergistic combinations was as follows: 31 out of 65 (48%) double combinations and 17 out of 21 (81%) triple combinations were synergistic. For a successful combination see Figure 1.

### Apoptotic effect of combinations at low doses in multiple myeloma models

Using FACS analysis, we investigated the beneficial (apoptotic) cell killing potential of the synergistic combinations at low concentrations looking for the ratio of apoptotic cells. We present concentration values predicted to produce maximal detectable cell killing based on the data extracted from viability assays on multiple myeloma cell lines and performed measurements for dose series that covered these values in order to identify the lowest value capable of achieving maximal cell death. In Figure 1, we show the apoptosis-inducing effect of selected successful combinations used on the LP1 (Figure 1A and 1B) and RPMI8226 cell lines (Figure 1C and 1D). The first combination (Figure 1B) targeted the CDK family, AKT1 and HDAC family driver targets of the LP1 myeloma cell line, while the second combination affected the CDK family, the MEK1/2 complex and NOTCH via gamma secretase. At a concentration of 0.122 nM, both combinations pushed the myeloma cells into early or late apoptosis in comparison to untreated controls.

### Solid tumor models

Parallel to the studies on myeloma we investigated the effectiveness of our method on solid tumor models using three adherent cell line models with well-established driver status: HCT116 and HT29 colon carcinoma cell lines and the A549 lung adenocarcinoma cell line.

### Targeting actually present versus absent driver genes

Prior to the combination studies we conducted an experiment on the HCT116 cell line to explore the difference between the effectiveness of the compounds of the DriverHit Library on the actually present driver-related proteins and molecules having no target in the given model. According to this, the first group of targeted proteins were products of driver genes associated with HCT116, whereas the second cohort of targets were not involved in the pathologic proliferation of HCT116 cells. We performed an unpaired *t*-test using the pIC_50_ values of the compounds. The two-tailed *P*-value turned out to be 0.0015. This indicates that prior to drug selection a careful analysis of the actually present and targetable driver genes has to be performed.

### Combinations used on colon cancer cell lines

To determine and characterize driver genes in HCT116 and HT29 colon cell lines we used the COSMIC database. Subsequently, we designed 77 for HCT116 and 82 drug combinations for HT29. Of the 77 HCT116 combinations 37 contained two and 40 had three drugs. Forty three combinations out of 77 proved to be synergistic (56%). Three out of 43 synergistic combinations (7%) had a CI value < 0.1. Sixteen of 37 double combinations (43%) and 27 of 40 triple combinations (68%) were synergistic. Out of the 82 combinations for HT29 55 had two and 27 had three drugs. Using CI_95_ as the basis for evaluation of synergism between compounds we found that 57 combinations out of 82 were synergistic (70%). Ten out of 57 synergistic combinations (18%) had a CI value < 0.1 representing an extremely strong synergism. The number of synergistic combinations was 31 (56%) in double and 26 (96%) in the case of triple combinations. Figure 1 shows synergistic combinations for each cell line. For the full data set for HCT116 and HT29 see Supplementary Table 1D and 1E.

### Driver-targeted combinations in the NSCLC model

In order to increase the diversity of models we also performed experiments on the A549 lung adenocarcinoma cell line. Out of 55 combinations for A549 28 contained two and 27 had three drug compounds. Forty six out of 55 combinations proved to be synergistic (84%). Twenty out of 46 synergistic combinations (43%) had CI values < 0.1. The number of synergistic combinations depending on the number of drugs included was 23 (82%) in the case of double and 23 (85%) in the case of triple combinations. For all combinations see Supplementary Table 1F. A representative combination is shown in Figure 1.

### Synergistic combinations on multiple myeloma patient-derived surviving cultures

For our experimental purposes we produced balanced patient-derived myeloma-stroma cell co-cultures and performed *in vitro* combination treatments in 9 cases (the balanced co-culture models were developed by F. Uher who passed the know-how to Vichem Ltd., unpublished results). Samples that were transformed to stable co-cultures originated from patients before or under treatment. We focused on already approved drugs or compounds being still in clinical trials. We used bortezomib and MG-132 as proteasome inhibitor monotherapy controls therefore the combinations used were comparable with the clinically applied therapies. Combinations were used at 0.5 and 0.5 μM concentrations and proteasome inhibitors were applied at 1 μM. We managed to effectively shift the cell killing profile from stromal cells to myeloma cells in most cases. A selection of our results is shown in Table 2. Corresponding monotherapy results are shown in Supplementary Table 2.

**Table 2:** Patient-derived surviving culture combination therapies

Patient No.	Drug combinations [0.5 μM + 0.5 μM]	Targets	Drivers	Myeloma cells			Stromal cells		
				Expected Inh %	Meas. Inh %	Ratio	Expected Inh %	Meas. Inh %	Ratio
P 1	GSK2126458 + Danusertib	PI3K-mTOR; AURKA; AURKB	PTEN, TP53	20.8	40.5	1.95	5.3	14.9	2.81
P 1	MG-132	proteasome			10.1			6.8	
P 2	GSK2126458 + Danusertib	PI3K-mTOR; AURKA;	PTEN, TP53	36	51.4	1.43	10.3	10.3	1
P 2	Danusertib + CUDC907	AURKA; HDACs	MLL3, ASXL1, TP53	32.8	43.4	1.33	18.4	18	0.98
P 2	Danusertib + Dinaciclib	AURKA; CDKs	CDKN2A, CEBPA, TP53	27	31.1	1.15	15.2	39.3	2.59
P 2	MG-132	proteasome			16.3			16.7	
P 3	GSK2126458 + Danusertib	PI3K-mTOR; AURKA; AURKB	PTEN, TP53	24.6	40.5	1.65	5.7	35.2	6.23
P 3	CUDC907 + Danusertib	HDACs; AURKA; AURKB	MSH2, MLL3, TP53	22.15	29.1	1.31	6.2	23.2	3.74
P 3	MG-132	proteasome			10.9			16.8	
P 5	Nintedanib + MG-132	FGFR2; FGFR3; PDGFR; VEGFR2; FGFR; VEGFR; PDGFR; proteasome	FGFR3, PDGFRA, TP53	32.8	47.4	1.45	37.8	32.5	0.86
P 5	Dinaciclib + Nintedanib	CDKs; FGFR2; FGFR3; PDGFR; VEGFR2; FGFR; VEGFR; PDGFR	FGFR3, PDGFRA, TP53	30.95	40.5	1.31	32.7	61.6	1.88
P 5	MG-132	proteasome			45.9			71.4	
P 6	Nintedanib + Bortezomib	FGFR2; FGFR3; FLT3; PDGFR; VEGFR2; proteasome	FGFR3, PDGFRA, TP53	20.1	40.4	2.01	41.2	14	0.34
P 6	Bortezomib	proteasome			33			79.1	

Expected Inh %: average of the combined drugs Inh % in monotherapy. Measured Inh%: percentage of dead cells detected. Ratio: ratio of the Expected Inh % and the Measured Inh %. In case of the ratio was higher than 1.1, the measured value was considered better than that expected so the combination was considered to be synergistic. If the ratio is less than 0.9, the measured value was considered worse than the expected; the combination is considered antagonistic. If the ratio ranges from 1.1–0.9, the combination performed as expected. Control rows contain Inh %s of the reference proteasome inhibitors in monotherapy for patient-derived co-cultures.

## DISCUSSION

Myeloma cell lines and patient-derived surviving cultures are multiple myeloma models, while HCT116 and HT29 are colon and A549 is lung adenocarcinoma cell lines. As a result, we intend to discuss the two groups separately due to their distinctive driver gene characteristics.

### Synergistic driver targeting combinations in multiple myeloma models

**Figure 3:**
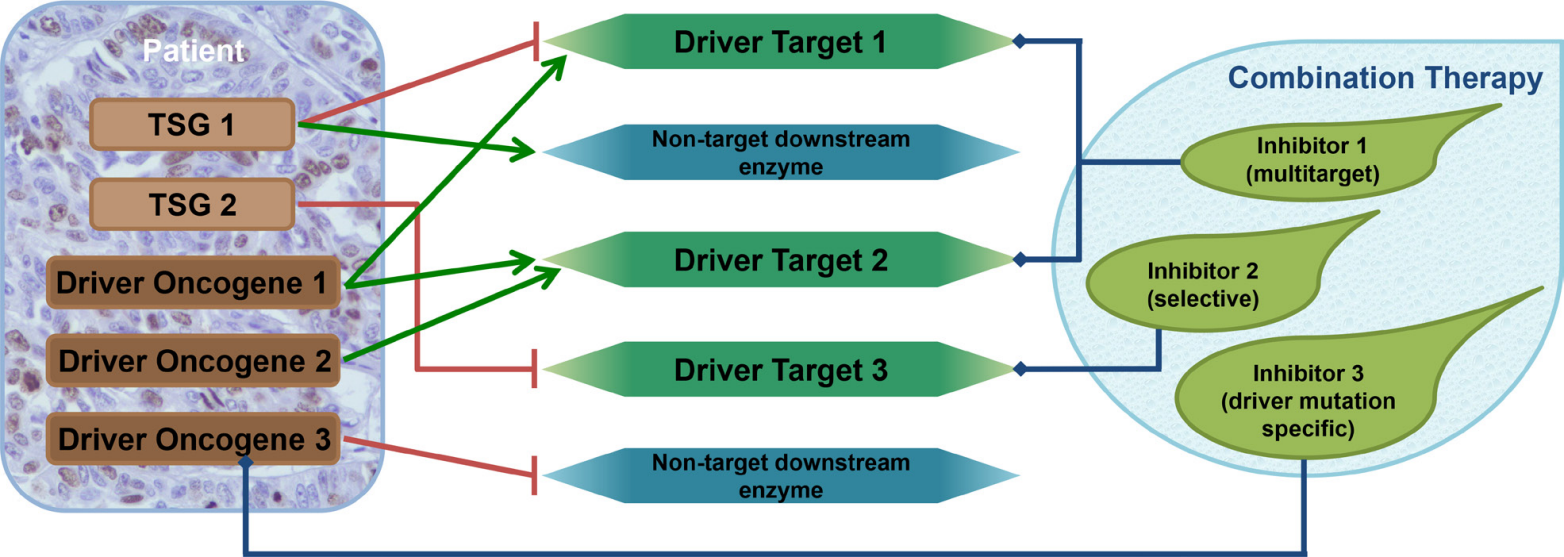
Combination therapy design using the driver gene concept. Tumors usually contain 2–8 driver genes. Tumor suppressor genes (TSG) harbor loss-of-function mutations, while oncogenes possess gain-of-function mutations. Oncogenes can be inhibited directly or via the downstream partners that they stimulate. TSG functional loss may be corrected by inhibiting the downstream partners they are supposed to inhibit naturally. These downstream elements are the driver targets. Although exact protein-selective small molecule inhibitors exist the majority of them have multiple protein targets. The diverse selectivity profiles of the inhibitors and the fact that different driver genes can share their driver targets indicate that more than one driver gene can be blocked by each compound. This confirms our finding that tumors with more than three driver genes can be handled using a mixture of merely three compounds.

Recent developments in myeloma treatment have led to new and more effective therapeutic protocols. Approved drugs constantly increase, the growing number of newly discovered actionable targets and the enhanced efficacy of novel compounds pave the way to prolonged survival and better quality of life. Similarly, to other tumors, it is becoming evident that targeting only one driver-related pathogenic protein probably never lead to cure but will merely generate therapy resistance and failure. Therefore, a careful assembly of potentially effective drug combinations based on a precise detection of a vulnerable targets will be necessary to improve our treatment results. In our *in vitro* experiments we found that blocking 2–5 cancer pathways using only 2–3 drugs was sufficient to reach maximal levels of cell killing at extraordinarily low doses due to synergisms among drugs (Figure 3).

We tested drug combinations in order to identify synergistic drug combinations for myeloma cell lines and surviving cultures. We found that drug combinations targeting driver gene-related targets tend to be synergistic and we determined novel targets for use together with the canonical ones. These new potent targets, not yet investigated in the context of multiple myeloma, were revealed as targets through identification of the specific driver genes and their related proteins.

Modern treatment of multiple myeloma is based on five pillars: a) immunomodulatory drugs (IMiDs) (i.e. lenalidomide, pomalidomide and thalidomide); b) proteasome inhibitors (i.e. bortezomib, MG-132 and carfilzomib); c) traditional cytotoxic drugs (i.e. doxorubicine); d) HDAC inhibitors (i.e., vorinostat or panobiostat) and e) corticosteroids (e.g., dexamethasone and prednisone). CDK inhibitors have also been extensively studied in clinical trials. Because IMiDs, cytotoxics and steroids do not target the signal transduction pathways of cancer cells only HDAC, proteasome and CDK inhibitors were considered as targeted compounds in recent myeloma studies [67].

### Combinations of HDAC inhibitors

Combination of the HDAC inhibitor CUDC907 with the BCL2 inhibitor GX15-070 at extremely low doses resulted in synergism and total cell killing in all three cell lines. The combinations of CUDC907 with tipifarnib also proved to be successful, although this did not allow for dosages as low as those used with the HDAC plus BCL2 inhibitor combinations. When used as a combination partner, the androgen receptor (AR) inhibitor flutamide analogue also improved the therapeutic efficacy of CUDC907. HDAC and CDK inhibitors were also synergistic in all cell lines. CUDC101, another HDAC inhibitor was synergistic with Dp44mT, a topoisomerase inhibitor. This finding could be observed in all cell lines with the exception of U266. With a reference to the triple combinations, HDAC inhibitors tended to synergize with combinations containing a CDK inhibitor and a cell line-specific inhibitor, such as PF-03084014 (a NOTCH inhibitor), trametinib (a MEK1/2 inhibitor), 6H05 (an allosteric KRAS inhibitor), BI2536 (a PLK inhibitor), GSK2126458 (a PI3K-mTOR dual inhibitor) and MKK2206 (an allosteric AKT inhibitor) (See Supplementary Table 1A–1C). The HDAC inhibitor CUDC907 showed synergism when used with the AURKA inhibitor danusertib on several surviving cultures.

### Combinations of proteasome inhibitors

In the case of U266 and patient derived co-cultures we were able to successfully enhance the effect of proteasome inhibitors in combinations but we were not successful to find synergistic partners when experimenting with the LP1 cell line. We found only one synergistic partner (CUDC101, an HDAC inhibitor) in studies with the RPMI8226 cell line. However, we showed, that targeting HDACs, PLK1, KRAS, CDKs, AURKA and FGFRs may provide significant benefits in the improvement of IC_95_ values for proteasome inhibitors. The results of our experiments on surviving cultures demonstrate that these new targets improve myeloma cell killing rather than stromal cell killing.

### Combinations of CDK inhibitors

We found the inhibition of CDKs to be exceptionally beneficial in all investigated multiple myeloma models. Dinaciclib, a pan-CDK inhibitor, synergizes with the vast majority of driver target inhibitors. The most potent partners for CDK inhibitors in multiple myeloma are the HDAC inhibitors CUDC101 and CUDC907, the MEK1/2 inhibitor trametinib, the NOTCH inhibitor PF-03084014, the BCL2 inhibitors GX15-070 and ABT-263, the IDH1/2 inhibitor AGI-5180, the FLT3 and JAK2 inhibitor SB1317 and also the FGFR-targeting inhibitors XL999 and nintedanib. Dinaciclib was also part of synergistic duos and trios with inhibitors affecting the PI3K-AKT-mTOR pathway. Dinaciclib was synergistic with the PI3K-mTOR dual inhibitor GSK2126458 in experiments on the LP1 cell line and in patient-derived surviving cultures, as well as with AKT1 inhibitors MK2206 and PP242. Dinaciclib also synergized with the proteasome inhibitor MG-132 and the AURKA inhibitor danusertib. FACS results of experiments on the RPMI8226 and LP1 cell lines also confirmed that CDK inhibitors can play a key role in combination therapy.

### Novel driver targets identified in multiple myeloma

Our results raised new potential targets in multiple myeloma therapy. These targets may offer effective therapeutic surfaces for use in combination with previously known targets. These new targets to be considered in multiple myeloma treatment are the NOTCHs, BCL2, the IDHs, AR, NF2, Porcupine, the JAKs, MEK1/2 and the Aurora kinases.

### Synergistic combinations in solid tumor models

The HCT116 and A549 cell lines both have activating KRAS mutations, while KRAS in HT29 is not mutated. We used farnesyl transferase inhibitors (lonafarnib and tipifarnib), allosteric KRAS inhibitors and downstream MEK1/2 inhibitors to eliminate the effect of mutated KRAS. The most successful targets and combinations covered the HDACs, the CHEKs, the PI3K-mTOR axis and BCL2 in all three cell lines. However, A549 has a wild-type TP53 and was therefore less sensitive to the blockage of TP53 TSG targets and more sensitive to the MALT1 inhibitor and JNK inhibitor 1. Results of combination therapies for these models are shown in Supplementary Table 1D (HCT116), 1E (HT29) and 1F (A549). With regard to the PI3K-AKT-mTOR pathway, PI3K and the PI3K-mTOR dual inhibitors were more effective than the AKT1 inhibitors. These measurements suggest that AKT1 inhibition could successfully complements PI3K-mTOR inhibition. The pan-HDAC and PI3K inhibitor CUDC907 and the BCL2 inhibitor GX15-070 were highly potent when applied as monotherapies because each of them targets 2–5 drivers at the same time.

## MATERIALS AND METHODS

For information on Materials and Methods see Supplementary Text 1.

## CONCLUSIONS

Concentrating on the individual driver gene pattern of a tumor may contribute to an effective selection method to design synergistic combination therapies. In our *in vitro* models we demonstrated that combination therapies based on the individual driver gene patterns are more efficient than monotherapies in the majority of cases. We concluded that strong synergisms among compounds in driver-targeted drug combinations may result in shorter treatment periods and/or may help to reduce unwanted toxic side effects, because it may allow us to use remarkably lower doses. Because of the common targets in drivers matched with the different inhibitory profiles of the particular drugs used, even double and triple combinations may be highly effective in extremely low concentrations. In each case we proposed crucial driver combinations which affect 3–5 important cancer pathways in a given system. By using the method of targeting tumor specific driver gene sets we would like to form a common rationale for combination therapy design in different types of cell lines or patient derived primary cultures.

## SUPPLEMENTARY MATERIALS




