# Supplementary material for: Targeted drug combination therapy design based on driver genes
Source: Oncotarget. 2019 Sep 3;10(51):5255–66. doi: 10.18632/oncotarget.26985 (PMC6731102; doi:10.18632/oncotarget.26985)
Supplement: Supplementary file 1 [file oncotarget-10-5255-s001.pdf]

Targeted drug combination therapy design based on driver genes

SUPPLEMENTARY MATERIALS

MATERIALS AND METHODS

Driver gene identification

In order to identify the potential driver gene set in the given cell lines we used two main data sources: the results of the Multiple Myeloma Cell Line Characterization Project [22] and the COSMIC database [23].

First, we adapted Vogelstein's driver gene theory and filtered the cell line-specific mutational data for the 138 potential driver genes. Then we looked for possible driver mutations in these genes and identified the mutations that were previously known for their protein activity alterations. Silent mutations were excluded. To classify novel or unknown genetic alterations as either activating or deactivating mutations we performed predictions considering the proximity of the mutations to the active sites in order to find out whether they had a possible effect on protein function or not. As a simplified approach, we considered that a mutation in the catalytic domain of the given oncogene enzyme or a mutation that directly affects the active site of an oncogene may be an activating mutation even if not previously described as such in the literature. In the case of tumor suppressor genes (TSGs), we considered that: 1. frame-shift mutations, 2. mutations in the catalytic or regulatory sites and 3. premature termination codon-causing mutations to be loss-of-function mutations. If the novel or unknown mutation were likely to have an effect on protein function, and we were able to determine the direction of this effect, we considered the potential driver gene carrying the mutation as a real driver gene in our system.

We obtained the mutational profile for the patient-derived multiple myeloma surviving cultures by sequencing. The molecular profile of myeloma cells was analyzed with next-generation sequencing (Ion Personal Genome Machine, Thermo-Fisher), using the 50-gene Ion AmpliSeq Cancer Hotspot Panel or the 409-gene Comprehensive Cancer Panel (Thermo-Fisher). Samples were sequenced in duplicate (two barcodes). Only variants present at greater than 2% in both analyses were considered as variants for further analysis.

Target identification

If driver oncogenes have enzyme activity their corresponding proteins can be inhibited directly by small-molecule inhibitors. If the oncogenes are not targetable (e.g. due to a lack of targeted inhibitors) the nearest downstream stimulated enzyme was targeted.

In the case of TSGs we cannot target the loss-of-function mutated gene's protein product so we chose to identify the downstream elements of the cancer pathway and target the proteins that are naturally inhibited/suppressed by the TSG which may start acting as "oncogenes" in these cases. We preferred to inhibit the closest downstream proteins with enzymatic activity.

When the TSG is also a transcription factor (TF) we identified the list of potential targets by manual literature mining to maximize the confidence level of target selection. Targets which were not related directly to the cancer pathways were excluded [26].

Drug identification

In order to have an easily accessible compound library against the driver genes and their related targets we created the DriverHit Library, which is a subset of the Nested Chemical Library™ (NCL) [27] and consists of published reference inhibitors as well as unpublished and patentable drug-like substances. The DriverHit Library has been built up around the 138 known cancer-driver genes utilizing the target identification method described above. For each driver gene we attempted to identify at least one target molecule. Recently, we have been able to target 114 driver genes out of 138 either directly or via related target proteins. We have been able to block all 12 cancer pathways at at least one point of the signal chain. Fifty-seven of the 114 driver genes are oncogenes and 57 are TSGs. Our search of the literature allowed us to define 181 targets that were related directly or indirectly to these drivers. Once we identified these targets we collected reference inhibitors synthesized their derivatives and novel structures and selected corresponding compounds from NCL based on similarity to the reference inhibitors and biochemical activity profiles. Out of the 181 targets

defined, 143 thus far are targetable by small-molecule inhibitors. The DriverHit Library currently contains 386 reference compounds which are mainly in clinical practice or development and 1236 analogous compounds and 1304 compounds were selected based on biological activity. As a result, the total number of compounds is 2926 at the time of this study, but we continue adding new compounds to the library.

The criteria for selecting the inhibitors for the combination therapy studies were as follows: 1. strong evidence that the inhibitor directly and successfully interacts with the given target protein, 2. a well-known inhibitory profile that matches most closely with the target set of a given model, 3. compounds with beneficial toxicity data from clinical trials or *in vivo* experiments were preferred but a documented and strong inhibitory effect on given targets was the most important factor for the decision to use a compound on cell lines. In the case of surviving cultures we focused more on approved drugs and compounds already investigated in clinical trials.

Cell lines and cellular viability assays

RPMI8226, U266 and LP1 cell lines were kindly provided by Innsbruck Medical University, while the HCT116, HT29 and A549 cell lines were purchased from ATCC (LGC Standards, Teddington, UK). HCT-116, HT29, A549, RPMI8226 and U266 cells were cultured in RPMI-1640 medium (Sigma-Aldrich, St. Louis, MO). The LP-1 cell line was cultured in IMDM, supplemented with 10% heat-inactivated fetal bovine serum (Sigma–Aldrich, St. Louis, MO) and 1% Ab/Am (Sigma-Aldrich, St. Louis, MO) at 37° C in a humidified incubator containing 5% CO_2.

For cell viability measurements we used the CellTiter–Glo Luminescent Cell Viability Assay Kit (Promega Co., Madison, WI). The luminescence signal was detected by Infinite M1000 Pro Multimode Reader (Tecan Group Ltd., Männedorf, Switzerland). All compounds were dissolved in DMSO; 1000 cells per well were plated in 30 μL in 384-well flat-bottom plates (PerkinElmer Inc., Waltham, MA). Experimental data were gained from a 10-point serial dilution of the compounds (dilution range varied from 30–0.00001 μM). Cell viability was measured after 72 hours. The ratio of the number of surviving cells and the number of cells in the untreated samples (positive control) was determined. Two types of negative control were employed for normalization: medium without cells containing 10 μM DMSO and cells treated with staurosporine at a final concentration of 20 nM. IC_{50} curves were constructed using XLfit curve-fitting add-in software (IDBS) for Microsoft Office Excel.

The agents used for the treatments were either purchased or provided by Vichem Ltd. Rigosertib was purchased from Allichem LLC (Baltimore, MD); BI2536 from BioBlocks Inc. (San Diego, CA); MK2206 and ABT-263 from ChemieTek (Indianapolis, IN); AGI-5198, GSK2126458, CUDC907, KW2449, lonafarnib, trametinib, PKI587, XL019, PP242 and tivantinib from Haoyuan Chemexpress Corporation (Shanghai, China); vorinostat, SGI-1027, 6H05, PF-03084014, carfilzomib, AGI-6780 and a stock of tipifarnib from MedChem Express (Monmouth Junction, NJ); staurosporine from Lead Discovery Center GmBH (Dortmund, Germany); another stock of tipifarnib and MG-132 from SelleckChem (Houston, TX); oltipraz from Santa Cruz Biotechnology, Inc. (Dallas, TX); Dp44mT from Sigma Aldrich (St. Louis, MO); NF279 from Tocris Bioscience (Bristol, UK); and GX15-070 from Wonda Science (Changzhou, China). The following compounds were synthesized by Vichem Ltd.: hesperadin, dinaciclib, perifosine, sorafenib, KRAS Inhibitor 12, MALT1 Inhibitor MI-2, JNK Inhibitor 1, SB1317, XL999, danusertib, XL765, nintedanib, AG879, IWP-2, CUDC101, the flutamide analogue specified as "Vichem flutamide analogue" in this study and a proprietary inhibitor specific to FGFR2 and FGFR3 denoted as "Vichem FGFR inhibitor".

The unpaired *t*-test used for the HCT116 monotherapy comparison was carried out using GraphPad Software (GraphPad Software, Inc., La Jolla, CA).

Surviving culture formation, maintenance and treatment

Cultures were derived from bone marrow samples collected from patients of the United Szent Istvan and Szent Laszló Hospital, Budapest, Hungary. Samples were handled anonymously. Each culture was maintained in DMEM and F12K medium (Gibco; 1:1 ratio) supplemented with 10% FBS. Fresh samples underwent a whole mononuclear cell isolation procedure (Ficoll–Pague PLUS, GE Healthcare, Little Chalfont, UK). The PBMC layers were placed in cell culture flasks until stabilization of a constant myeloma–stromal cell ratio that was usually reached after 4 weeks of maintenance. Subsequently, co-cultures were transferred to 6- (later to 12-) well plates (VWR, Radnor, PA) with an equal number of cells in each well. A portion of samples were sent for sequencing in order to determine the affected driver gene set for each patient. Monotherapies and combination therapies were performed in parallel. The amount of cells and thus the available number of wells were different in each case. Monotherapies and the combination therapies were performed using inhibitors at a low dose (1 μM) so that the differences in inhibition percentages (Inh%) were clearly distinguishable. The agents were dissolved in DMSO and pure DMSO was used as control. The final concentration of DMSO was 0.02% in each well. It is important to note that there was no technical opportunity for parallel measurements so potential standard deviation could only be deduced from the standard deviation of the controls. For future studies, we intend to double the number of wells used for each patient sample. After an

incubation period of 72 hours we determined the number of living and dead myeloma and stromal cells in each culture by flow cytometry, separating the cells based on light scattering (stroma/myeloma) and propidium–iodide (PI) dying (living/dead).

FACS measurements on the multiple myeloma cell lines

The numbers of living, dead and apoptotic cells were determined by Fluorescence-Activated Cell Sorting (FACS) analysis using Annexin V-FLUOS and PI double-staining. In brief, cells were centrifuged after drug treatment and resuspended in 1× binding buffer containing 2.5 mM of calcium (Ca^{2+}). Cells were incubated with Annexin V-FLUOS (Roche Diagnostics GmbH, Mannheim, Germany) and PI (Sigma–Aldrich, St. Louis, MO) in the dark at room temperature for 20 minutes. Next, samples were reconstituted with 1× binding buffer and subjected to flow cytometry using a FACSCalibur (BD Bioscience, Franklin Lakes, NJ) to analyze the populations using CellQuest Pro software (BD Bioscience). Gated cells were analyzed using FL1H and FL2H log scale histograms. Data were presented as percentages of cells.

Statistical methods

We performed the measurements on cell lines in 2–12 repetitions. For determination of the IC_{50}, CI_{50}, IC_{95} and CI_{95} values we used CompuSyn software (ComboSyn Inc., New York, NY). This software program calculates the Combination Indices via the Chou-Talalay method, which is the most reliable and widespread approach to determine whether two or more compounds are acting synergistically [28, 29]. In this study, we present cellular IC_{50} and IC_{95} values and the corresponding Combination Indices (CI_{50} and CI_{95}) in order to more accurately characterize the effects of the compounds on the studied cell lines. In contrast to pharmacological publications that almost exclusively refer to IC_{50} values we consider the IC_{95} and the corresponding CI_{95} values to be more representative as our aim was to eliminate all cancer cells, even the resistance-causing cancer stem cells. In cases of $1 > CI_{95}$ values drug combinations were considered to be synergistic and approaching the maximal cell-killing effect.

Supplementary Table 1A: Marked as Supplementary Table 1A that contains data obtained from experiments on the RPMI8226 multiple myeloma cell line. See Supplementary_Table_1

Supplementary Table 1B: Marked as Supplementary Table 1B which contains the data obtained from experiments on U266 multiple myeloma cell line. See Supplementary_Table_1

Supplementary Table 1C: Marked as Supplementary Table 1C which contains data obtained from the experiments on LP1 multiple myeloma cell line. See Supplementary_Table_1

Supplementary Table 1D: Marked as Supplementary Table 1D which contains data obtained from the experiments on HCT116 colon cell line. See Supplementary_Table_1

Supplementary Table 1E: Marked as Supplementary Table 1E which contains data obtained from the experiments on The HT29 colon cell line. See Supplementary_Table_1

Supplementary Table 1F: Marked as Supplementary Table 1F that contains data obtained from the experiments on the A549 lung cell line. See Supplementary_Table_1

Supplementary Table 2: Effect of various drug monotherapies on patient-derived, surviving myeloma-stroma cell co-cultures

Patients	Drugs in 1 µM	Targets	Myeloma Inh %	Stroma Inh %
Patient 1	GSK2126458	PI3K-mTOR	37.2	8.5
Patient 1	Danusertib	Aurora; AKT	4.3	2.1
Patient 2	GSK2126458	PI3K-mTOR	50.1	10.3
Patient 2	Dinaciclib	CDKs	32.2	20
Patient 2	CUDC907	HDAC	43.7	26.5
Patient 2	Danusertib	AURKA; AKT	21.8	10.3
Patient 3	GSK2126458	PI3K-mTOR	30.1	7.4
Patient 3	CUDC907	HDAC	26	8.5
Patient 3	Danusertib	AURKA; AKT	19.1	3.9
Patient 5	Nintedanib	FGFR2; FGFR3; PDGFR; VEGFR2	19.7	4.2
Patient 5	Dinaciclib	CDKs	42.2	61.2
Patient 5	CUDC907	PI3Ka; HDAC	54	46.6
Patient 5	MG-132	proteasome	45.9	71.4
Patient 6	Nintedanib	FGFR2; FGFR3; PDGFR; VEGFR2	6.8	3.3
Patient 6	Dinaciclib	CDKs	27.2	51.3

Fraction of dead myeloma and stroma cells (Inh%) were assessed by flow cytometry. Values are the result of one parallel experiment.
